# Direct observation of knock-on reaction with umbrella inversion arising from zero-impact-parameter collision at a surface

**DOI:** 10.1038/s42004-021-00453-x

**Published:** 2021-02-12

**Authors:** Matthew J. Timm, Lydie Leung, Kelvin Anggara, John C. Polanyi

**Affiliations:** Lash Miller Chemical Laboratories, Department of Chemistry, University of Toronto, Toronto, ON Canada

**Keywords:** Surface chemistry, Physical chemistry, Reaction kinetics and dynamics, Surface spectroscopy

## Abstract

In Surface-Aligned-Reactions (SAR), the degrees of freedom of chemical reactions are restricted and therefore the reaction outcome is selected. Using the inherent corrugation of a Cu(110) substrate the adsorbate molecules can be positioned and aligned and the impact parameter, the collision miss-distance, can be chosen. Here, substitution reaction for a zero impact parameter collision gives an outcome which resembles the classic Newton’s cradle in which an incident mass ‘knocks-on’ the same mass in the collision partner, here F + CF_3_ → (CF_3_)′ + (F)′ at a copper surface. The mechanism of knock-on was shown by Scanning Tunnelling Microscopy to involve reversal of the CF_3_ umbrella as in Walden inversion, with ejection of (F)′ product along the continuation of the F-reagent direction of motion, in collinear reaction.

## Abstract (web summary)

Collision geometries are hard to control, making transition states in chemical reaction hard to study. Here the authors show that a linear transition state leads to formation of reaction product ‘knocked-on’ along the continuation of the direction of reagent approach.

## Introduction

With the advent of quantum mechanics^1^, it became evident that partially-formed bonds could stabilize the transition state, facilitating chemical reaction. Using this formalism H. Eyring and M. Polanyi plotted the potential energy surface for a collinear reaction H+H_2_→H—H—H→H_2_+H, in which the intermediate configuration was the transition state stabilized by the presence of collinear extended H—H bonds^2^.

Early attempts to gain experimental information concerning the geometry of transition states began with the scattering of directed gaseous reagent atoms from dipolar molecules aligned in electric fields^3–5^. Important insights into reaction dynamics between gaseous molecules were obtained from crossed molecular beams, however, these studies averaged over all impact parameters, precluding control of the collision geometry^6^. The adsorption of reagents on a surface has allowed the collision geometry to be varied by “Surface-Aligned-Reaction”, SAR^7–9^. Scanning tunneling microscopy (STM) has provided direct information on molecular alignment in surface reactions^10–13^. A recent extension of SAR employing STM achieved a high degree of directionality in reactive collisions, employing the ordered surface-atoms as a collimator of energetic reagent “projectiles”^14,15^. These projectiles were formed by electron-induced dissociation of chemisorbed precursor molecules so that the projectiles collided with fixed chemisorbed “target” molecules at selected impact parameters, *b*. The impact parameter is defined as the perpendicular distance to the center-of-mass of the target molecule at the closest approach^14^.

In the present work, the projectile is an F-atom, the target a chemisorbed CF_3_ and the selected impact parameter *b* = 0 ± 0.5 Å. Here STM shows that an efficient substitution reaction yields a product (F)′-atom “knocked-on” from the CF_3_ along the continuation of the collision-direction. The CF_3_ is shown, directly by STM, to invert its CF_3_ umbrella as the reagent F displaces the product (F)′ in the collinear “knock-on” reaction. Substitution with retention of collision-direction in the transition state indicates that the dynamics involve one-dimensional reaction, as proposed in early work on the classic substitution reaction, H+H_2_→H_2_+H, and in the widely used concept of Walden inversion^16^.

## Results and discussion

### Alignment on the surface

**Fig. 1:**
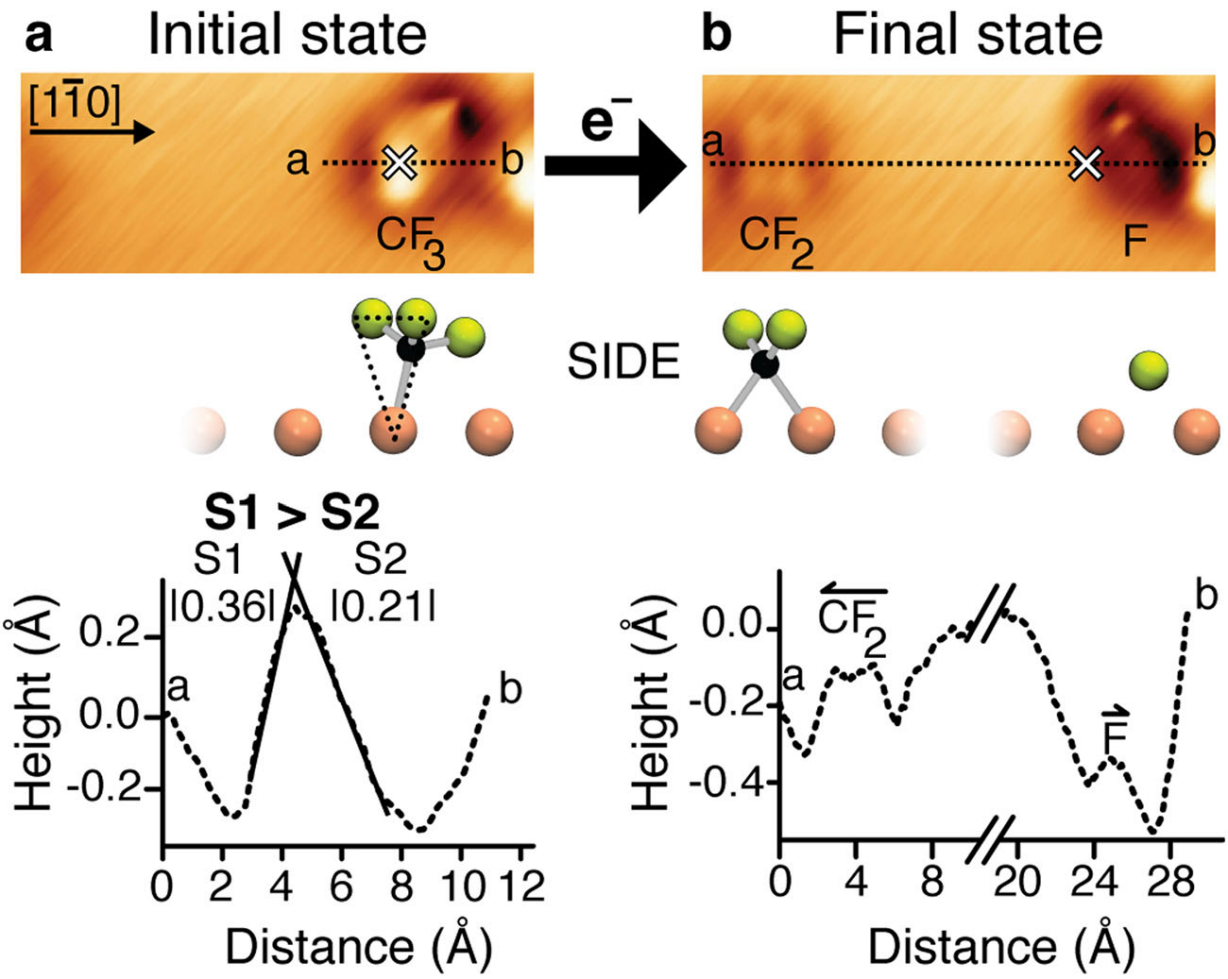
Electron-induced dissociation of chemisorbed CF_3_ gives directed CF_2_ and F. **a** The high-contrast STM image (30 Å × 12 Å, *I* = 1.0 nA, *V* = −5 mV) of a single CF_3_ chemisorbed at the Cu(110) surface, in which the backside of the CF_3_ is shown to face to the left of the image, is highlighted by a black dashed triangle in the side view. The height-profile of the CF_3_ reagent is taken along [1$\bar 1$0] from **a** to **b**. The slopes indicated on this line profile are S1 = |0.36 ± 0.02| Å/Å and S2 = |0.21 ± 0.01| Å/Å. **b** The electron-induced dissociation of the CF_3_ shows long-range recoil of the dissociation product CF_2_ to the left and a short-range recoil of F to the right of the white cross in the STM image, both along a [1$\bar 1$0] copper row. The height-profile of the two products, **a**–**b**, is taken along [1$\bar 1$0]. The white cross overlaid upon each STM image marks the initial position of the CF_3_ prior to dissociation.

The adsorption and reaction of individual chemisorbed CF_3_ molecules at a Cu(110) surface at 4.6 K has been previously examined by STM and density functional theory (DFT) calculations^14,15^. The CF_3_ molecule binds atop a Cu atom through its C-atom, giving a tetrahedral configuration (Fig. 1a). Two of the C–F bonds are tilted away from the copper surface by 31°, termed the raised CF_2_ end of CF_3_. The third C–F bond is tilted only 10° out of the surface plane, pointed along [1$\overline 1$0]. This almost in-plane C–F bond is seen by STM to be the one that breaks in electron-induced reaction^14^. The backside of the CF_3_ adsorbate is composed of the raised CF_2_ and the Cu atom below, as highlighted by the black dashed triangle in the side view of Fig. 1a. The F reagent will be shown to attack the backside of CF_3_, which opens as the CF_3_ inverts.

High-contrast imaging of a single chemisorbed CF_3_ at 4.6 K, obtained by scanning at a bias close to the Fermi level with a functionalized STM tip^17–21^, is shown in Fig. 1a (and Supplementary Fig. 1a). The distinction between the CF_2_ and CF ends of CF_3_ is visible in the height-profile of Fig. 1a, from a to b along the [1$\overline 1$0] direction (see Supplementary Note 1). The measured slope on the left-hand side of the height-profile, S1, is steeper than that on the right-hand side, S2. The steeper slope (S1: |0.36| Å/Å) corresponds to the backside of the CF_3_ adsorbate (triangle, Fig. 1a).

The electron-induced dissociation of CF_3_ yields a difluorocarbene (CF_2_) 18.0 Å to the left of the CF_3_ reagent position (shown by a white cross in Fig. 1) and an F-atom 3.7 Å to the right along the [1$\overline 1$0] direction (Fig. 1b). These recoil distances agree with those previously reported for the two products^15^. The locations of the CF_2_ and F-atom products from the CF_3_ dissociation show that the alignment of the CF_3_ umbrella is correctly assigned with the raised CF_2_ end at the left.

### Electron-induced reaction

**Fig. 2:**
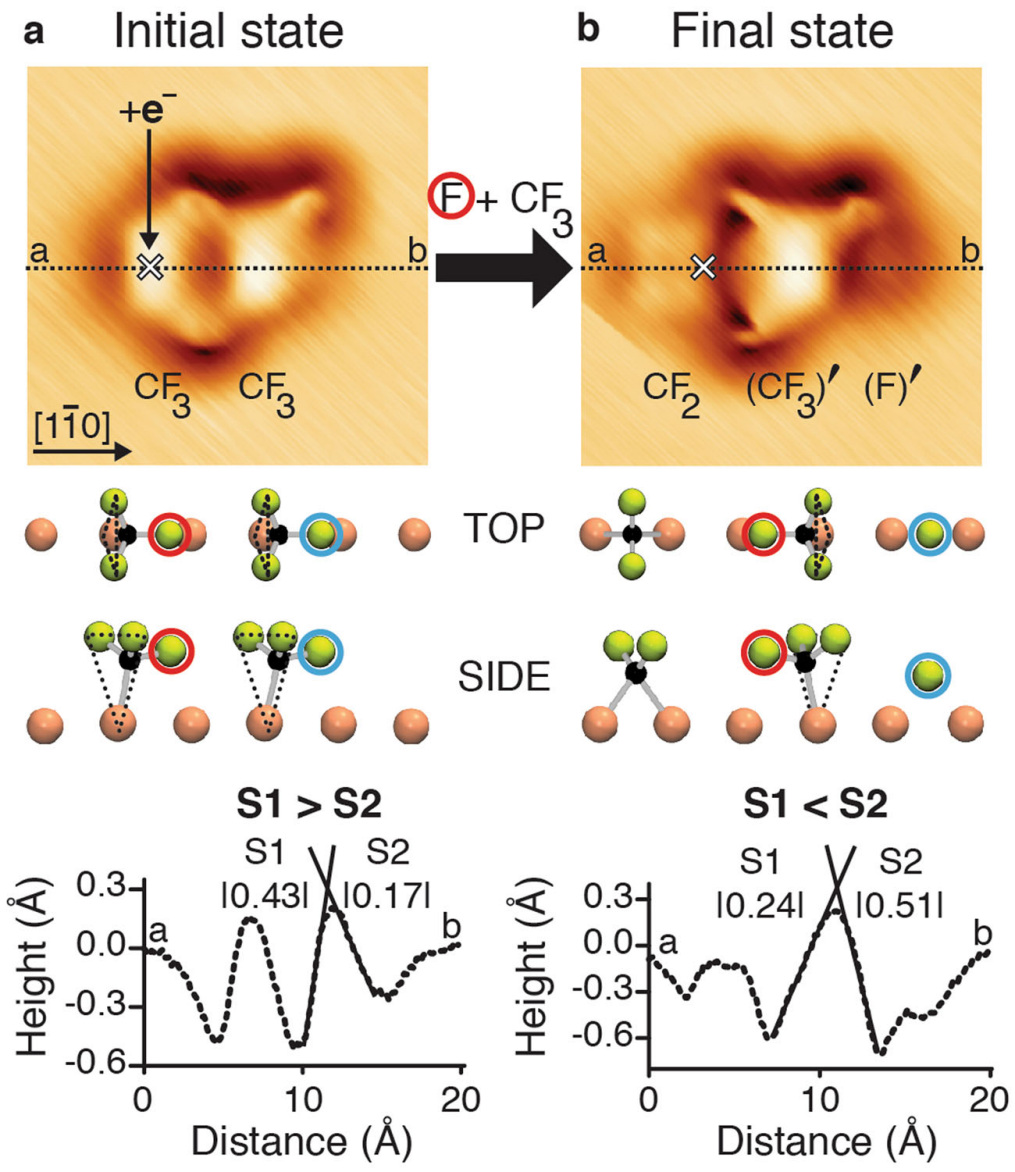
“Knock-on” reaction: F plus CF_3_ gives (CF_3_)′ plus (F)′. **a** The STM image (20 Å × 20 Å, *I* = 0.1 nA, *V* = −1 mV) of the initial state of a pair of CF_3_ molecules, chemisorbed at the Cu(110) surface, before electron addition to the CF_3_ “precursor“ molecule at the left (location marked by the white cross). In the side view, below, both CF_3_ are oriented with their backsides at the left (black dashed triangles). In the top and side views (lines two and three), the F-atom at the tip of the CF_3_ umbrella of the precursor is circled in red and in blue for the CF_3_ target. **b** The final state STM image (20 Å × 20 Å, *I* = 0.1 nA, *V* = −1 mV) shows the three products of the electron-induced reaction: CF_2_, (CF_3_)′ and (F)′. In the top and side views, the CF_3_ and (CF_3_)′ are seen to have an inverted umbrella. The height-profiles (bottom panels) are measured along [1$\bar 1$0] from **a** to **b**. The measured slopes of the CF_3_ target in **a** are S1 = |0.43 ± 0.02| Å/Å and S2 = |0.17 ± 0.01| Å/Å. Those of the (CF_3_)′ in **b** are S1 = |0.24 ± 0.01| Å/Å and S2 = |0.51 ± 0.01| Å/Å.

The “knock-on” reaction is initiated by electron-induced dissociation of a chemisorbed CF_3_ (Fig. 2). The CF_3_, shown at the left in Fig. 2a, is termed the “precursor” of CF_2_ and of F. The atomic F, coming from CF_3_, recoils along the Cu-row and collides with a second chemisorbed CF_3_, the ‘target’ at the right in Fig. 2a, to give an inverted CF_3_ umbrella (CF_3_)′ and a knocked-on F-atom (F)′. The initial state comprised a pair of chemisorbed CF_3_ on the same Cu-row, separated by 5.17 ± 0.06 Å (Fig. 2a). The adsorption configuration shown in Fig. 2a was the only one observed for a pair of CF_3_. The asymmetry identified in the height-profile of a single CF_3_ is also observed in the a–b height-profile of each CF_3_ of the pair. The CF_3_ precursor and CF_3_ target exhibit the same tilted configuration, with the backside at the left (Fig. 2a). This implies that the in-plane C–F bond of the CF_3_ precursor is directed toward the backside of the CF_3_ target molecule. This adsorption configuration of the CF_3_ pair agrees with the simulated STM image obtained from the calculated geometry (Supplementary Fig. 2a).

Electron addition to the CF_3_ precursor gave three products, chemisorbed CF_2_, (CF_3_)′, and (F)′ on the same Cu-row (Fig. 2b and Supplementary Fig. 2b). As in Fig. 1, the CF_2_ and F-atom products are shown to the left and right sides of the initial position of the CF_3_ reagent. The F-atom travels 8.94 ± 0.04 Å, seemingly passing through the chemisorbed CF_3_ target. The (F)′ is formed as the result of a zero impact parameter collision between the F-atom reagent and the CF_3_ target. The F-atom reagent has displaced an (F)′-atom product from the target (the displaced product atom is highlighted by the blue circle in Fig. 2). The (F)′ product recoiled along the continuation of the direction of approach of the F-atom reagent, hence being designated as “knocked-on”. Concurrently, the (CF_3_)′ is displaced 1.0 Å to the left of the prior CF_3_ target position, with its umbrella inverted compared with its original alignment in Fig. 2a. This umbrella inversion is clearly evidenced by the reversed S1:S2 ratio in the height-profile (from S1 < S2 to S1 > S2).

**Fig. 3:**
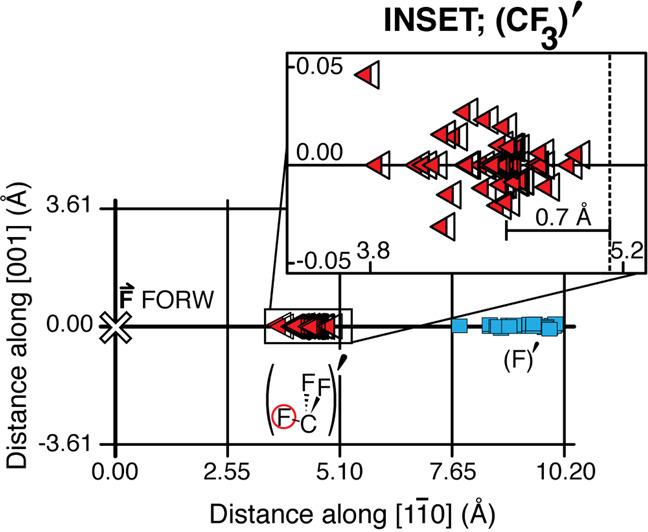
Product distribution, (CF_3_)′ and (F)′, from knock-on reaction with umbrella inversion. Final positions of the products of reaction, following collision of the reagent F-atom with the adjacent CF_3_ target. The reagent F-atom was formed by electron-induced dissociation of a CF_3_ precursor. The two reaction products are chemisorbed (CF_3_)′ shown as red-tipped triangles, and (F)′ product shown as blue squares. A schematic of the inverted (CF_3_)′ with the F-atom circled in red is shown below in the figure. The scale corresponds to the unit-cell dimensions of the Cu(110) surface. An enlarged view of the distribution of the (CF_3_)′ products is shown in the inset. The lateral spread in the inset is < ± 2 % of the distance between the rows. The location of the CF_3_ target is marked by a dashed line. The (F)′ product (blue squares) is found on the copper row, along with the continuation of the ‘forward’ direction of the incoming F-atom marked ‘$\overrightarrow {\mathrm{F}}$ FORW’.

Knock-on reaction is found for 46 of the 71 cases of F + CF_3_ collision (65 ± 10%). Figure 3 shows the observed positions of the knock-on reaction products, (CF_3_)′ and (F)′, relative to the initial position of the CF_3_ precursor (white cross). The data are folded such that the initial state is the same as in Fig. 2, with the origin at the location of the CF_3_ precursor. Dissociation of the CF_3_ precursor yields two oppositely recoiling products, CF_2_ and F. We define the recoil direction of the F-atom reagent as the “forward” direction ($\overrightarrow {\mathrm{F}}$ FORW). As the CF_2_ recoils backward, we did not consider it to be a part of the knock-on reaction. This CF_2_ is found to recoil a wide variety of distances along the Cu-row, uncorrelated with knock-on. The distance distribution of all the reaction products is shown in Supplementary Fig. 3a. A striking feature of the (CF_3_)′ and (F)′ knock-on products is that they are collinear with the incoming reagent F. This indicates that the linear momentum of the projectile is retained throughout the existence of the transition state.

From the CF_3_ precursor position, the (CF_3_)′ product (red-tipped triangles in Fig. 3) is observed at an average distance of 4.50 ± 0.07 Å and the (F)′ product (blue squares) at 9.44 ± 0.08 Å. An enlarged view of the (CF_3_)′ distribution is given in the inset to Fig. 3. For each case of knock-on reaction the (CF_3_)′ is displaced slightly backwards from the CF_3_ target position (dashed line). The displacement is found to be 0.67 ± 0.07 Å on average, and invariably coincides with inversion of the CF_3_ umbrella in the target. This displacement is observed in each case of inversion, where the STM resolution allows the umbrella alignment to be obtained (50% of cases). The second product of knock-on reaction, (F)′ was found to have traveled ~4.27 Å past the CF_3_ target, along the continuation of the direction of the F-atom reagent.

In a minority of cases (25 out of 71 cases: 35 ± 7 %), a different outcome is observed; “unsuccessful” knock-on (Supplementary Fig. 3b and Supplementary Note 2). The F-atom reagent is found to have scattered in the backward direction from the CF_3_ target, rather than forward. In this “unsuccessful” knock-on the CF_3_ target was not observed to undergo inversion, nor was it observed to be displaced from its initial position.

**Fig. 4:**
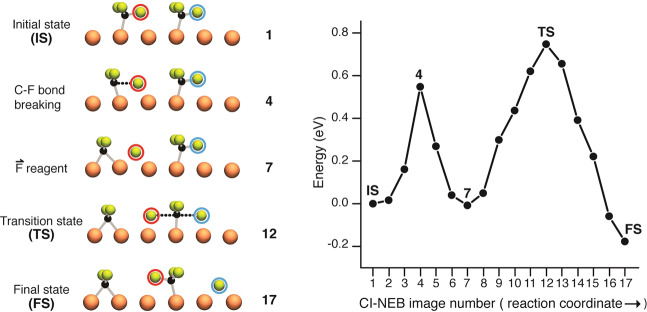
Climbing image-nudged elastic band (CI-NEB) pathway for knock-on reaction. Minimum-energy pathway across a DFT potential energy surface as obtained from a CI-NEB calculation. Five representative CI-NEB images along the pathway are shown at the left of the figure. In the initial state, IS, the CF_3_ precursor is at the left and the CF_3_ target is at the right. The F-atom, originating in the CF_3_ precursor, is marked by a red circle, whereas the (F)′ product is marked by a blue circle.

Previous studies of electron-induced dissociation of CF_3_ have reported, in addition to travel along a Cu-row, lateral travel of F-atom products to an adjacent Cu-row. This was attributed, through molecular dynamics calculations, to in-plane rotation of the intact CF_3_. Computation showed that a rotation of < 7° was sufficient to account for the observed lateral travel of the F-atom^15^. A rotation of the CF_3_ precursor by 7° increases the impact parameter to *b* = 0.5 Å, providing a valuable upper limit for the miss-distance that permits knock-on reaction. The fact that *b* > 0.5 Å, which favors orbital motion, gives no observable knock-on product, indicates that knock-on is not due to orbiting of the projectile around the target. Instead, the system provides a direct path (Fig. 4), over a low energy barrier, leading from incoming F to out-going (F)′.

### Knock-on mechanism

To obtain the mechanism of collision between the reagent F-atom and the CF_3_ target at zero impact parameter, a climbing image-nudged elastic band (CI-NEB) calculation was performed^22,23^. The initial and final states correspond to those observed by STM in Fig. 2. From this CI-NEB calculation, the successive configurations of the reagents are identified as the system progresses along the reaction coordinate.

The knock-on reaction shown in Fig. 4 takes place across two successive barriers. The first barrier involves the dissociation of the CF_3_ precursor to produce CF_2_ and an F-atom—the latter circled in red. This first barrier of 0.55 eV is required for the extension of the in-plane C–F bond (image 4). Following C–F bond breaking, the CI-NEB shows CF_2_ and F at the nearest adsorption sites. For the F-atom this is the short-bridge site beneath, whereas for the CF_2_ fragment it is the short-bridge site 1.6 Å away (image 7).

The second barrier of the CI-NEB results from the energized F-atom projectile (red) colliding with the CF_3_ target, and thereafter ejecting an (F)′ (blue). The barrier for this F-atom substitution is 0.76 eV required to form the symmetric linear transition state, F—CF_2_—(F)′ (Fig. 4, image 12). This arises from F attacking the backside of the chemisorbed CF_3_ target, as in Walden inversion^16^. Following this, the CF_3_ umbrella completes its inversion and knocks-on an (F)′-atom, which is repelled to its final position 3.7 Å away (Fig. 4, images 13–17). The small computed barrier, ~1 eV, accords with the high probability of 65% observed for knock-on. The mechanism derived from the CI-NEB supports knock-on occurring through a linear transition state with collinear ejection of (F)′ along the continuation of the direction of the incoming projectile.

## Conclusions

In this work, we have examined the novel realm of “zero impact parameter” (zero collision miss-distance) chemistry, as an innovative means to control the scattering of reaction products at metal surfaces. We have shown that reaction at zero impact parameter occurs for the most part collinearly in one-dimension (1D), while inverting the target molecule as in classic Walden inversion, seen directly here. Notably, the reaction products are expelled collinearly with the reagent motion in what we term “knock-on” dynamics. The new concept is that reduced dimensionality in reagent motion leads, through momentum conservation, to reduced dimensionality in product motion. The appearance of 1D reaction is that an energized reagent, the “projectile”, has passed through the “target” molecule to emerge collinearly on the other side. The consequence for chemistry is that the reagent energy can be passed on in successive collinear collisions, leading to a “knock-on chain”. In a world where energy is to be conserved, these are favorable dynamics. This resembles the conservation of linear momentum that underlies the motion in Newton’s cradle, knocking on a succession of steel balls.

## Methods

### STM

Experiments were performed with an LT-UHV Scanning Tunneling Microscope (Omicron) at 4.6 K. The Cu(110) surface was prepared by repeated cycles of Argon sputtering and annealing to 800 K. Tungsten STM tips were electrochemically etched in 3M NaOH solution. Functionalization of the STM tip was accomplished by positioning the tip above clusters of CF_3_ and I-atom adsorbates and repeatedly performing −5 to −10 V pulses without identifying the species transferred to the tip. Functionalization of the tip was confirmed when the apparent height of the chemisorbed CF_3_ was 3× greater than that obtained for a non-functionalized tip at the previous imaging conditions^14,15^. All STM images were taken at 4.6 K using constant current mode with the bias referred to the sample. CF_3_ was obtained by dosing trifluoroiodomethane, CF_3_I, onto the cleaned Cu(110) at 79.5 K, which dissociated upon adsorption. Then the crystal was cooled down to 4.6 K in the STM stage. The electron-induced dissociation of CF_3_ was performed by (i) placing the tip over the adsorbate, (ii) adjusting the tip height, (iii) turning off the feedback loop, and (iv) ramping the sample bias to *V*_pulse_ (between 1.3 and 1.8 V). Dissociation was confirmed by imaging the products in a subsequent scan. The distance and direction of the dissociation products were analyzed using the WSxM Software^24^.

### DFT calculations

The DFT calculations were performed using the Vienna Ab initio Simulation Package (VASP)^25,26^ on the SciNet supercomputer Niagara cluster^27,28^. The calculations used the projector augmented wave method^29,30^ and the generalized gradient approximation (GGA) with the Predew-Burke-Ernzerhof functional^31^. Grimme’s semi-empirical dispersion correction was added to correct for Van der Waals interactions^32^. The energy cutoff for the plane-wave basis set was 400 eV. The Cu(110) surface was modeled by a (3 × 10) slab consisting of 150 Cu atoms in five layers, separated with a 17 Å vacuum layer. All atoms were allowed to move except for the bottom two layers of Cu atoms, which were frozen. The adsorption geometries were obtained by relaxing the system until the force on each unfrozen atom was >0.01 eV/Å. The relaxation calculations were performed using a single Γ-point *k*-mesh sampling. Molecular structures were visualized using the Visual Molecular Dynamics software^33^. Simulated STM images and height-profiles were obtained through calculations using the Tersoff-Hamann approximation^34^, and visualized using the Hive software^35,36^.

### CI-NEB calculations

The CI-NEB calculations were performed in VASP based on DFT using Γ-point only. Between the initial and final states calculated from the experimental data shown in Fig. 2, a local minimum was found along the reaction coordinate. This local minimum corresponds to CI-NEB image 7. This local minimum was relaxed following the same procedure as the initial and final states. To determine the barrier between the initial state and the local minimum, we used five images. A total of nine images were used to find the barrier between the local minimum and the final state. The calculations were conducted until the forces orthogonal to the band were <0.02 eV/Å. The Fast Inertial Relaxation Engine optimizer^37^ was used to minimize the forces. To achieve convergence a time step of 0.01 (in 10.18 fs units) was used, instead of the time step previously employed^38^.

## Supplementary information

Supplementary Information

## Data Availability

The data that support the findings of this study are available from the corresponding author upon reasonable request.
