# Supplementary material for: Direct observation of knock-on reaction with umbrella inversion arising from zero-impact-parameter collision at a surface
Source: Commun Chem. 2021 Feb 12;4:14. doi: 10.1038/s42004-021-00453-x (PMC9814886; doi:10.1038/s42004-021-00453-x)
Supplement: Supplementary file 1 — Supplementary Information [file 42004_2021_453_MOESM1_ESM.pdf]

Supplementary Information

Direct observation of knock-on reaction with umbrella inversion arising from zero-impact-parameter collision at a surface

Matthew J. Timm†, Lydie Leung†, Kelvin Anggara and John C. Polanyi*

†These authors contributed equally.

Lash Miller Chemical Laboratories, Department of Chemistry, University of Toronto, 80 St. George Street, Toronto, Ontario M5S 3H6, Canada

*e-mail: john.polanyi@utoronto.ca

Table of contents

Supplementary Figures;
Supplementary Fig. 1 to Supplementary Fig. 3

Supplementary Notes;
Supplementary Note 1 and Supplementary Note 2

Supplementary Figures

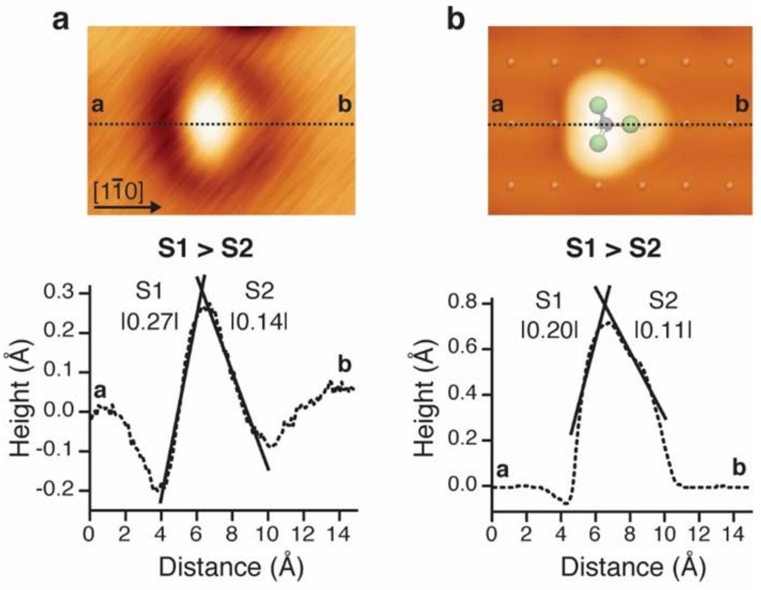

Supplementary Figure 1 | Comparison of experimental and simulated (computed) STM images and height-profiles (a-b) for chemisorbed CF_3. a, the STM image (15 Å × 11 Å, *I* = 1.0 nA, *V* = -5 mV) and height-profile obtained for chemisorbed CF_3 on Cu(110). The height-profile was taken along the $[1\bar{1}0]$ direction from a to b. **b,** Simulated STM image and height-profile computed from the calculated adsorption geometry of CF_3. The height profile was taken along the $[1\bar{1}0]$ direction from a to b. The measured slopes for the CF_3 molecule shown in (a) are S1 = |0.27 ± 0.01| Å/Å and S2 = |0.14 ± 0.01| Å/Å.

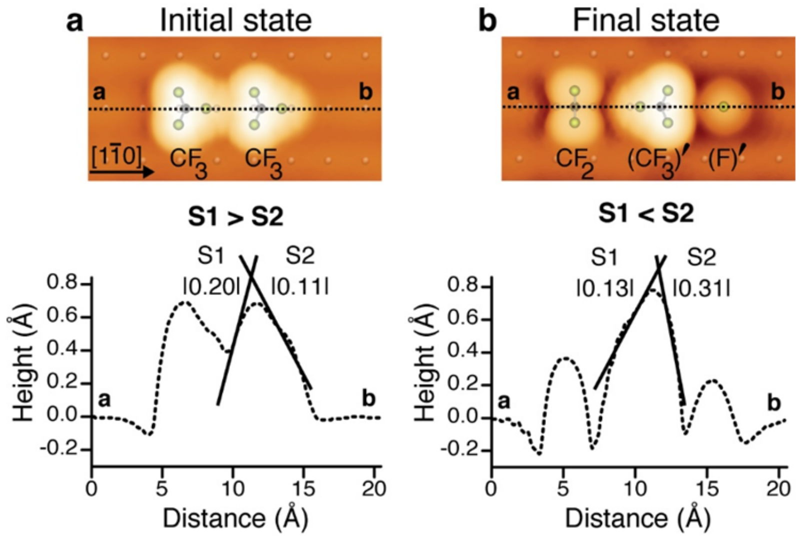

Supplementary Figure 2 | Calculated images and height-profiles for the initial and final states of the knock-on reaction. a, The simulated STM image of the initial state of two adjacent CF_3 adsorbate molecules shown in Fig. 2 of the main text (with calculated adsorption geometry overlaid). As indicated in the line-profile for the right-hand CF_3 (the 'target'), a slope of S1 > S2 shows that the adsorbate sits with its raised CF_2 (backside) facing to the left. **b,** The simulated STM image of the final state in Fig. 2, with overlain top view of the three products of the electron-induced reaction: CF_2, $(CF_3)'$ and $(F)'$. The $(CF_3)'$ is seen in the top view to have an inverted umbrella, as is apparent from the inversion of the calculated slopes to give S1 < S2. The height-profiles in the lower panels were taken along the $[1\bar{1}0]$ direction, from a to b.

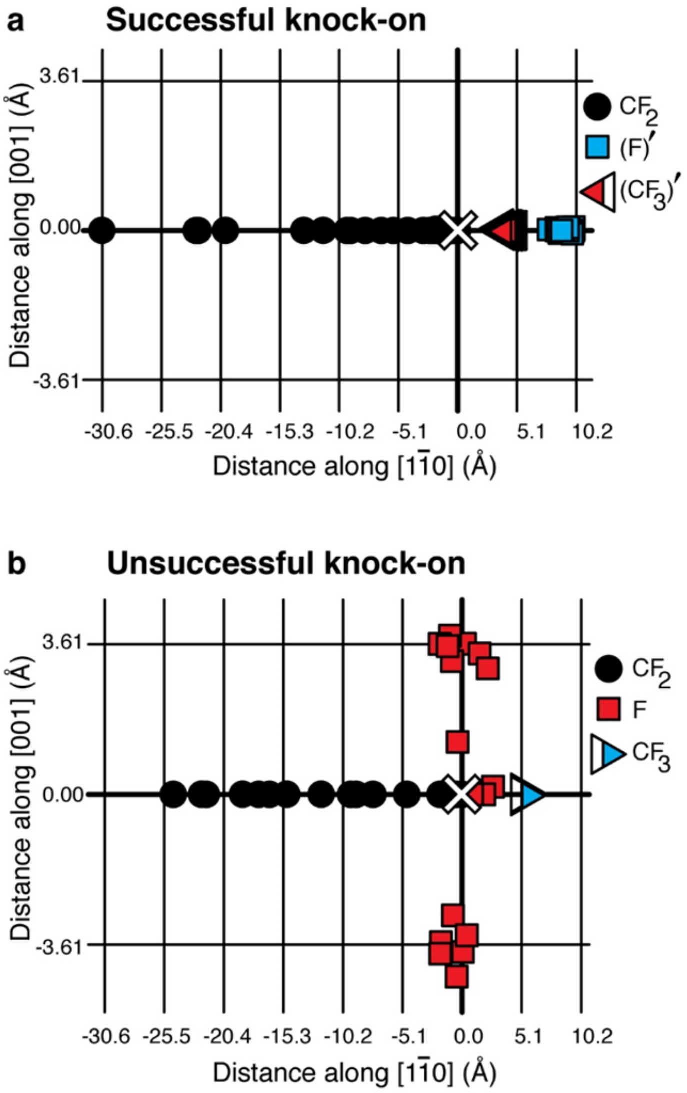

Supplementary Figure 3 | Product distribution (CF_2, $(CF_3)′$, (F)′ above; and CF_2, CF_3, F below) observed after the electron-induced dissociation of the left-hand CF_3 in an adjacent pair of CF_3 (Fig. 2a and Supplementary Fig. 2a). a, The distribution of CF_2, $(CF_3)′$ and (F)′ products for 46 cases of *successful* knock-on reaction. **b,** The distribution of CF_2, CF_3 and F products for 25 cases of *unsuccessful* knock-on. The white cross in both panels indicates the position of the CF_3 precursor position.

Supplementary Notes

Supplementary Note 1:

Supplementary Fig. 1 shows that there is agreement between the experimental and simulated images obtained for an isolated chemisorbed CF_3. The simulated image was obtained from the computed geometry of a chemisorbed CF_3. The height-profiles for both images have a similar S1 > S2 difference in slope, supporting our assignment of the raised CF_2 (left side of the simulated image) and in-plane C-F bond of the CF_3 (right side of the simulated image).

Supplementary Note 2:

The most striking difference in Supplementary Fig. 3 is successful knock-on of an (F)′-atom in the first instance (Supplementary Fig. 3a) and no reaction (‘unsuccessful knock-on’) of the F-atom reagent in the second instance (Supplementary Fig. 3b).

For successful knock-on, the F-atom is found beyond the CF_3 target, along the $[1\bar{1}0]$ direction, whereas for unsuccessful knock-on the F-atom is scattered backward from the CF_3 target. In the majority of unsuccessful knock-on (22 of 25 cases) the F-atom reagent has scattered to an adjacent Cu-row, surmounting a 0.9 eV diffusion barrier. For the minority of unsuccessful knock-on (3 out of 25 cases) the F-atom is trapped in a well located at the short-bridge site between the CF_3 precursor and the CF_3 target positions. This well corresponds to the local minimum obtained from CI-NEB calculation (Fig 4, image 7).

For successful knock-on, the CF_3 target is slightly displaced due to its umbrella inversion, whereas for unsuccessful knock-on the CF_3 target does not invert nor therefore change position. The recoil distribution on CF_2 product does not correlate with the outcome of knock-on reaction, showing that it is a spectator to knock-on.
